# Validity of a cardiology fellow performance assessment: reliability and associations with standardized examinations and awards

**DOI:** 10.1186/s12909-022-03239-4

**Published:** 2022-03-15

**Authors:** Michael W. Cullen, Kyle W. Klarich, Kristine M. Baldwin, Gregory J. Engstler, Jay Mandrekar, Christopher G. Scott, Thomas J. Beckman

**Affiliations:** 1 Department of Cardiovascular Medicine, Mayo Clinic, 200 First St. SW, Rochester, Minnesota 55905 USA; 2 Department of Information Services, Mayo Clinic, 55905, 200 First St. SW, Rochester, Minnesota USA; 3 Department of Health Sciences Research, Division of Biomedical Statistics and Informatics, Mayo Clinic, 200 First St. SW, Rochester, Minnesota 55905 USA; 4 Division of General Internal Medicine, Department of Internal Medicine, Mayo Clinic, 200 First St. SW, Rochester, Minnesota 55905 USA

**Keywords:** Assessment, Cardiology fellowship, Evaluation, Training, Validity evidence

## Abstract

**Background:**

Most work on the validity of clinical assessments for measuring learner performance in graduate medical education has occurred at the residency level. Minimal research exists on the validity of clinical assessments for measuring learner performance in advanced subspecialties. We sought to determine validity characteristics of cardiology fellows’ assessment scores during subspecialty training, which represents the largest subspecialty of internal medicine. Validity evidence included item content, internal consistency reliability, and associations between faculty-of-fellow clinical assessments and other pertinent variables.

**Methods:**

This was a retrospective validation study exploring the domains of content, internal structure, and relations to other variables validity evidence for scores on faculty-of-fellow clinical assessments that include the 10-item Mayo Cardiology Fellows Assessment (MCFA-10). Participants included 7 cardiology fellowship classes. The MCFA-10 item content included questions previously validated in the assessment of internal medicine residents. Internal structure evidence was assessed through Cronbach’s α. The outcome for relations to other variables evidence was overall mean of faculty-of-fellow assessment score (scale 1–5). Independent variables included common measures of fellow performance.

**Findings:**

Participants included 65 cardiology fellows. The overall mean ± standard deviation faculty-of-fellow assessment score was 4.07 ± 0.18. Content evidence for the MCFA-10 scores was based on published literature and core competencies. Cronbach’s α was 0.98, suggesting high internal consistency reliability and offering evidence for internal structure validity. In multivariable analysis to provide relations to other variables evidence, mean assessment scores were independently associated with in-training examination scores (beta = 0.088 per 10-point increase; *p* = 0.05) and receiving a departmental or institutional award (beta = 0.152; *p* = 0.001). Assessment scores were not associated with educational conference attendance, compliance with completion of required evaluations, faculty appointment upon completion of training, or performance on the board certification exam. R^2^ for the multivariable model was 0.25.

**Conclusions:**

These findings provide sound validity evidence establishing item content, internal consistency reliability, and associations with other variables for faculty-of-fellow clinical assessment scores that include MCFA-10 items during cardiology fellowship. Relations to other variables evidence included associations of assessment scores with performance on the in-training examination and receipt of competitive awards. These data support the utility of the MCFA-10 as a measure of performance during cardiology training and could serve as the foundation for future research on the assessment of subspecialty learners.

## Introduction

Cardiovascular diseases (CV) is the largest subspecialty of IM, with 2978 fellows enrolled in general cardiology fellowships during the 2019–2020 academic year [1]. Trainees in CV fellowships must acquire unique technical, cognitive, and procedural skills distinct among medical subspecialties [2, 3]. The validity of clinical assessments previously established for learners in general internal medicine may not apply to subspecialty fellowships, particularly CV fellows. Therefore, the validity of cardiology fellows’ assessments during fellowship training carries importance for both the CV and broader subspecialty medical education communities.

Establishing the validity of instrument scores is necessary for meaningful assessments [4–6]. An assessment is considered valid when the outputs or scores justify the given score interpretation [7]. Validity evidence originates from 5 sources: content, response process, internal structure, relations with other variables, and consequences [4, 5, 8]. The necessary contribution or weight of these evidence sources varies, depending on the instrument’s purpose and the stakes of the assessment.

Prior studies have examined validity evidence for assessments of internal medicine (IM) residents. For example, a multisource observational assessment of professionalism was associated with residents’ clinical performance, professional behaviors, and medical knowledge [9, 10]. Direct clinical observations of IM residents have been established as a valid measure of clinical skills [11]. However, research has demonstrated factor instability of the same teaching assessment when applied to general internists versus cardiologists, suggesting that one assessment method may vary across different educational environments and medical specialties [12]. Furthermore, very limited research exists on learner assessments among CV fellows [13–15].

In this validity study, we sought to investigate content, internal structure, and relations to other variables evidence for an assessment of CV fellows’ performance at the Mayo Clinic. The relations to other variables evidence examined associations between CV fellows’ assessment scores and key educational outcomes during cardiology fellowship training including in-training examination (ITE) scores, conference attendance, evaluation completion, cardiology certification examination failure, receiving an award, and faculty appointment upon completion of training. This study builds upon prior work from our group examining the association of application variables with subsequent clinical performance during cardiology fellowship training [15].

## Methods

### Setting and participants

This was a retrospective cohort study of 7 classes of cardiology fellows at the Mayo Clinic in Rochester, Minnesota beginning 2 years of core clinical training from July, 2007 to July, 2013 and thus completing clinical training from June, 2009 to June, 2015 [15]. During the study period, all fellows completed 2 years of required core clinical rotations followed by research and/or subspecialty training. All fellows entering the core clinical training program during this study period were eligible.

### Instrument development and validation

Validity evidence for this study was based on Messick and the current Standards for Educational and Psychological Testing which state that all validity pertains to the construct of interest and that evidence to support the construct comes from the following sources: content, internal structure, response process, relations to other variables (i.e., criterion), and consequences [4, 16]. This study utilized the Mayo Cardiology Fellows’ Assessment (MCFA-10) with 10 common items from faculty-of-fellow end-of-rotation assessment forms that were structured on a 5-point scale (1, lowest; 3, average; 5, highest i.e. “Top 10%”). Table 1 provides descriptions and sources of content evidence for each item in the MCFA-10. The content of these items reflected previously validated questions to assess IM residents at the Mayo Clinic. They demonstrated evidence of content, internal structure based on factor analysis and internal consistency reliability, and relations to other variables including performance on standardized tests and markers of professional behaviors [9, 10, 17–19]. These items also corresponded to questions used to assess the performance of cardiology fellows in our previous work [15]. Items comprising the MCFA-10 have been utilized at Mayo Clinic for formative reasons to provide feedback to residents and fellows to improve their performance and for summative purposes to assist with ranking and award decisions.

**Table 1:** Core items in the Mayo Cardiology Fellows’ Assessment (MCFA-10) with description and sources of content evidence

Item	Description	Sources of content evidence	Categories of validity evidence for the referenced sources
Accuracy and completeness of gathered information	Obtains thorough and appropriately comprehensive data from the history, physical exam, and medical record	Similar item demonstrated excellent internal structure reliability [17]; appropriate and effective patient care is a component of core ACGME patient care competency [20]	Internal structure, content
Interpretation of patient data effectively	Synthesizes patient’s history, physical exam, laboratory data, and differential diagnoses	Similar item demonstrated excellent internal structure reliability [17]; appropriate and effective patient care is a component of core ACGME patient care competency [20]	Internal structure, content
Selection of appropriate diagnostic tests	Utilizes appropriate, cost effective, and evidence-based diagnostic testing	Similar item demonstrated excellent internal structure reliability [17]; appropriate and effective patient care is a component of core ACGME patient care competency [20]	Internal structure, content
Formulation of an effective plan and patient management strategy	Interprets test results accurately; demonstrates good clinical judgment	Similar item demonstrated excellent internal structure reliability [17]; appropriate and effective patient care is a component of core ACGME patient care competency [20]	Internal structure, content
Effective and concise case presentations	Clearly articulates the salient aspects of cases with both written and verbal communication	Similar item demonstrated excellent internal structure reliability [17]; effective exchange of information is a component of core ACGME interpersonal and communication skills competency [20]	Internal structure, content
Efficiency of patient work-ups	Prioritizes and conducts patient evaluations in a timely manner	Prior studies have identified the value of efficiency in clinical teaching [21]; efficient patient care is a component of core ACGME patient care competency [20]	Content
Quality of dismissal letter	Completes accurate, concise, and responsive documentation to referring providers	Item felt to be important given nature of Mayo Clinic referral practice and communication with external providers	Content
Professionalism	Accepts responsibility; demonstrates empathy, honesty, initiative, and integrity; practices mutual respect; places needs of patient first	Similar to component of validated assessment of professionalism [9﻿, 10]	Content, relations to other variables
Commitment to own education	Attends conferences; completes required readings; practices self-directed learning	Similar to component of validated assessment of professionalism [9, 10]	Content, relations to other variables
Systems based practice	Practices cost-effective care; references clinical guidelines; leverages system resources for the benefit of patients	ACGME requires assessment of competency in systems based practice [22]	Content

Abbreviations: *ACGME* Accreditation Council for Graduate Medical Education

### Relations to other variables evidence

Table 2 outlines independent variables used for relations to other variables evidence. In-training examination (ITE) scores were identified as the mean percent correct on the cardiology ITE taken annually during the first 3 years of fellowship [23]. Conference attendance was the number of departmental educational conferences attended during the 2 years of core clinical training. Fellows with satisfactory evaluation compliance completed ≥90% of their assigned rotation and faculty evaluations during their core clinical training. Board examination performance was classified as pass or fail on the American Board of Internal Medicine’s initial Cardiovascular Disease Certification Exam. Fellows receiving a staff appointment were those who joined the Mayo Clinic faculty upon completion of training. Receiving an award was defined as attaining ≥1 divisional, departmental, or institutional award during fellowship training.

**Table 2:** Definitions of variables to support relations to other variables evidence

Conference attendance	Number of fellowship sanctioned educational conferences attended during 2 years of core clinical training
Evaluation compliance	Completion of ≥90% of rotation and faculty evaluations during study period
Board exam performance	Pass / fail performance on the initial attempt on the American Board of Internal Medicine’s Cardiovascular Disease Certification Exam
Staff appointment	Faculty appointment at Mayo Clinic or affiliated sites upon completion of training
Receiving an award	Receipt of ≥1 competitive divisional, departmental, or institutional award during fellowship training
In-training examination score	Mean percent correct on the cardiology in-training examination

To develop relations to other variables evidence, faculty-of-fellow end-of-rotation scores including the MCFA-10 items were combined across each subject’s 2 years of core clinical training [15]. Individual items from each faculty’s assessment were averaged to obtain a total score for that particular assessment form. Scores from all assessments that a fellow received were averaged into one score on a continuous scale. The specific items in each assessment varied slightly over time and across rotations. However, the MCFA-10 items (Table 1) formed the foundation of the instrument under consideration.

### Data collection

This study received an exemption from the Mayo Clinic Institutional Review Board. All data were de-identified prior to analysis and were treated as confidential, in accordance with our standard approach to educational data. Data for this study pertained only to Mayo Clinic cardiology fellows and were the property of our cardiology fellowship program. External sources were not accessed to collect data for this study.

### Statistical analysis

We inspected the distribution of continuous variables prior to conducting the primary analysis to determine if they should be treated as normally distributed. We reported distributions of continuous predictor and outcome variables as mean ± standard deviation or median (interquartile range) as appropriate based on the distribution, and n (%) for categorical or ordinal variables. In subjects with missing ITE results, we imputed the median ITE score. We examined relationships between predictor and continuous outcome variables using simple and multiple linear regression methods. We built the multiple regression model based upon the results of the univariate regression models. Those variables with *p* < 0.1 on univariate regression were candidate variables in the multiple regression model. We then removed variables one at a time until all variables had *p* ≤ 0.05. We reported results of linear regression models as beta coefficients with corresponding 95% confidence intervals (CI) and presented regression R-squared values as a means of comparing strength of association of different variables and models. Pairwise associations between variables in the multiple regression model, including Pearson chi-square tests and Spearman correlations, were further evaluated for evidence of co-linearity. To support internal structure validity evidence, we calculated Cronbach’s α across all raters for the MCFA-10 items [4, 24]. The threshold for statistical significance was set at *p* ≤ 0.05. Analyses were conducted using SAS version 9.4 (Cary, NC).

## Results

### Participant characteristics

Of the 67 fellows who entered the 2 years of clinical training during our study period, 2 were excluded because they entered the fellowship through non-traditional means and did not have complete application data available. Therefore, the final study group included 65 fellows.

The median age of the study population at the beginning of clinical training was 30 years, and 41 (63%) were male. The fellows in this study trained at 18 different residency programs. Table 3 outlines the distribution of the variables in our study group.

**Table 3:** Distribution of variables to support relations to other variables evidence

Variable	Value^*^
Median age (years)	30
Male gender	41 (63)
In-training examination score^†^	69 ± 7
Conference attendance^‡^	97 ± 30
Evaluation compliance ≥90%	34 (52)
Board exam performance	
Pass	62 (95)
Fail	2 (3.1)
Not available	1 (1.5)
Staff appointment	23 (35)
Receiving an award	20 (31)

^*^Values are presented as mean ± standard deviation or n (%) of the 65 total study subjects, unless otherwise indicated

^†^ITE score was missing in 23 subjects and available in 42 of 65 subjects

^‡^Conference attendance data was missing in 1 subject

### Internal structure evidence

As noted in our prior work, 142 faculty evaluators completed 4494 fellow assessments containing a total of 27,941 individual evaluation items. The internal consistency reliability for the MCFA-10 items was excellent (Cronbach’s α = 0.98) [15, 25]. Of the 27,941 individual items, on a 1–5 ordinal scale, 23 were 1’s (0.1%), 293 were 2’s (1.1%), 4009 were 3’s (14.4%), 16,830 were 4’s (60.2%), and 6786 were 5’s (24.3%). The mean assessment score across all fellows during the first 2 years of clinical training was 4.07 ± 0.18.

### Relations to other variables evidence

Correlation analyses between the independent variables in the study demonstrated a positive association between ITE scores and conference attendance (Spearman Correlation Coefficient = 0.42; *p* = 0.006), a strong positive association between receiving an award and a Mayo Clinic staff appointment (75% of those who received an award joined the faculty vs. 18% of those who did not receive an award; *p* < 0.0001), and a weak positive association between evaluation compliance and conference attendance (mean attendance 104 conferences in those with ≥90% evaluation compliance vs. 88 conferences in those with < 90% evaluation compliance; *p* = 0.04). No other significant associations between independent variables in this study existed.

Table 4 demonstrates the results of the linear regression analysis to support relations to other variables evidence. Univariate analysis found that higher ITE scores and receiving a divisional, departmental, or institutional award were associated with higher aggregate assessment scores. Other predictor variables, including educational conference attendance, compliance with completion of evaluations, board examination performance, and staff appointment upon completion of training were not associated with aggregate assessment scores. In a multi-variable model adjusted for ITE score and receiving an award, both variables remained significantly associated with aggregate assessment scores that included the MCFA-10 items. Overall R^2^ for the multivariable model was 0.25.

**Table 4:** Linear regression analyses to support relations to other variables evidence

	Univariate analysis^a^		Multivariable analyses^a^	
Variable	β-estimate (95% CI)	*p*-value	β-estimate (95% CI)^b^	*p*-value
In-training examination score^c^	0.083 (0.002–0.164)	0.05	0.088 (0.003–0.153)	0.05
Conference attendance^d^	0.001 (− 0.001–0.002)	0.25		
Evaluation compliance (≥90%)	0.053 (−0.037–0.142)	0.25		
Board examination failure	−0.237 (− 0.495–0.019)	0.07		
Staff appointment	0.034 (−0.060–0.129)	0.48		
Receiving an award	0.164 (0.075–0.254)	< 0.001	0.152 (0.065–0.237)	0.001

^a^β-estimates are reported for the associations between the listed independent variable and each subject’s aggregated faculty-of-fellow end-of-rotation assessment scores that included MCFA-10 items

^b^β-estimates are adjusted for receiving an award, in-training exam score, and missing in-training exam score (*n* = 23)

^c^The association between in-training exam score and the primary outcome was examined in the 42 of 65 subjects where in-training exam score was available. For the remaining 23 subjects with unavailable ITE results, in-training exam score was imputed as the median score. β-estimates for both the univariate and multivariable analyses are reported for a 10-point increase in in-training exam scores

^d^Conference attendance data was missing in 1 subject

## Discussion

To our knowledge, this is the first validation study reporting multiple types of evidence for an instrument assessing clinical performance of cardiology fellows. We identified excellent internal consistency and strong evidence for relations to other variables, particularly ITE scores and receiving an award. We also provide evidence for content based on experience with prior instruments and published literature. These findings offer important implications for the assessment of cardiology fellows. They also extend our previous work regarding relationships between the cardiology fellowship application and clinical performance during subsequent training [15].

Current approaches assert that validity relates to the construct of interest and that evidence to support the construct comes from five sources: content, internal structure, response process, relations to other variables, and consequences [4, 5]. A systematic review of validation studies in the field of medical education revealed that most commonly reported sources of validity evidence come from the categories of content, internal structure, and relations to variables [26]. In this study, content evidence for the MCFA-10 comes from items with established validity evidence in other settings. Several components of the MCFA-10 directly relate to ACGME core competencies [20]. This study is among the first to establish content evidence of these items in subspecialty medicine fellowships, particularly CV. Our findings also demonstrate internal structure evidence for the MCFA-10 based on excellent internal consistency reliability (Cronbach’s α = 0.98).

A major component of validity evidence for MCFA-10 scores in this study comes from relations to other variables. We found that ITE scores and receiving an award are significantly associated with faculty-of-fellow clinical assessment scores that contain the MCFA-10 items. In contrast, we did not identify an association between assessment scores and conference attendance, evaluation completion, board certification, or staff appointment upon completion of training. These findings infer that cardiology faculty may emphasize medical knowledge and competitive achievement, which could be reflected in their performance assessments of the fellows. Additionally, variables such as conference attendance, evaluation completion, and board examination performance may be less likely to influence cardiology faculty-of-fellow assessments because these variables are less visible than displays of medical knowledge and receipt of awards.

The variables used for relations to other variables evidence in this study encompass important dimensions of assessment in medical education. ITE scores and board certification exam performance reflect standardized measures of medical knowledge. Receiving an award and staff appointment after finishing training reflect competitive achievement and career advancement, respectively. Evaluation completion and conference attendance are markers of professionalism. The correlations of assessment scores containing MCFA-10 items with ITE scores and receipt of awards suggest that our assessment captures elements of both medical knowledge and achievement. Although relationships between assessment scores and professionalism-related variables were less robust, items in the MCFA-10 have previously demonstrated associations with other assessments of professionalism [9, 10].

As noted above, prior work has shown factor instability of learner-of-faculty clinical teaching assessment scores between general medicine and cardiology environments [12]. This implies that validated assessments of internal medicine residents may not hold the same validity in cardiology fellows. Importantly, our study found that our MCFA-10 items, which have been validated in internal medicine residents, remain valid for assessing clinical performance in cardiology fellows [9, 10, 17–19]. This new association builds on prior literature and provides unique relations to other variables evidence. It suggests that, while the clinical learning environments between general internal medicine and cardiology differ, the MCFA-10 items work well to assess internal medicine residents and cardiology fellows and retain their validity across both subspecialties. Future work may examine if these MCFA-10 items maintain their validity in other institutions and medical specialties.

We performed exploratory analyses examining associations between independent variables in our study. We found positive correlations between (1) ITE scores and conference attendance, (2) receiving an award and staff appointment, and (3) evaluation compliance and conference attendance. The association between ITE scores and conference attendance has been documented in other settings among IM residents [27]. Previous work has demonstrate a strong association between the CV ITE scores and performance on the ABIM’s CV initial certification exam [28]. Our findings further support utility of the cardiovascular CV ITE as a marker of medical knowledge acquisition.

The identified correlation between receipt of awards and staff appointment likely reflects overall professional aptitude, as Mayo Clinic applies similar criteria to faculty selection as it does to recognizing recipients of prestigious awards. Finally, the correlation between evaluation compliance and conference attendance may indicate conscientious behaviors among CV fellows in this study, which has been previously documented among IM residents [10].

### Limitations

While this is a single institution study, the Mayo Clinic Cardiovascular Diseases Fellowship is one of the largest in the country, making it particularly suited for this research from the perspective of sample size and program representativeness. Furthermore, the knowledge, professionalism, and performance variables in our study are widely utilized by other CV training programs. For enhanced generalizability, we acknowledge that multi-institutional studies including diverse cardiology trainees and fellowship programs are necessary to validate our findings. Our study enrolled fellows who completed clinical training between June, 2009 and June, 2015 because were able to collect complete data on that cohort. Our findings remain relevant given the durable nature of the MCFA-10 items over time. 95% of fellows in our study passed their initial board examination, thus limiting the utility of this variable in regression analysis. We still demonstrated an association between ITE score and faculty assessment scores, supporting the association between MCFA-10 items and medical knowledge. Our study used linear regression to perform the relations to other variables analysis when most scores were 3’s, 4’s, or 5’s on a 5-point scale. This is a common practice in education research, particularly when data is symmetrically distributed around an elevated mean, as ours was [29]. Finally, although this study did not report response process or consequences validity evidence, it did report the most commonly sought sources of validity evidence in medical education, as previous research documented [26].

## Conclusions

We are unaware of previous validity studies on methods for assessing cardiology fellows. This study supports validity of MCFA-10 scores as an effective means of assessing clinical performance among cardiology fellows. This study should serve as a foundation for the assessment of cardiology fellows and for future investigations of the MCFA-10 in cardiology fellowship training.

## Data Availability

The datasets generated and analyzed during this study are not publicly available to maintain confidentiality of the study subjects. Inquiries regarding the availability of data in this study can be directed to the study’s first author, Dr. Cullen.

## Abbreviations

CI: Confidence interval
CV: Cardiovascular diseases
IM: Internal medicine
ITE: In-training examination
